# The Protective Effect of *Staphylococcus epidermidis* Biofilm Matrix against Phage Predation

**DOI:** 10.3390/v12101076

**Published:** 2020-09-25

**Authors:** Luís D. R. Melo, Graça Pinto, Fernando Oliveira, Diana Vilas-Boas, Carina Almeida, Sanna Sillankorva, Nuno Cerca, Joana Azeredo

**Affiliations:** 1 Laboratório de Investigação em Biofilmes Rosário Oliveira, Centre of Biological Engineering, University of Minho Braga, 4710-057 Braga, Portugal; gracapinto@ceb.uminho.pt (G.P.); fernandoefoliveira@ceb.uminho.pt (F.O.); dianavilasboas@ceb.uminho.pt (D.V.-B.); carina.almeida@iniav.pt (C.A.); sanna.sillankorva@inl.int (S.S.); nunocerca@ceb.uminho.pt (N.C.); 2 INIAV, IP—National Institute for Agrarian and Veterinary Research, Vairão, 4485-655 Vila Do Conde, Portugal; 3 INL-International Iberian Nanotechnology Laboratory, Av. Mestre José Veiga, 4715-330 Braga, Portugal

**Keywords:** phage, biofilms, biofilm matrix, phage/host interactions, *S. epidermidis*

## Abstract

*Staphylococcus epidermidis* is a major causative agent of nosocomial infections, mainly associated with the use of indwelling devices, on which this bacterium forms structures known as biofilms. Due to biofilms’ high tolerance to antibiotics, virulent bacteriophages were previously tested as novel therapeutic agents. However, several staphylococcal bacteriophages were shown to be inefficient against biofilms. In this study, the previously characterized *S. epidermidis*-specific *Sepunavirus* phiIBB-SEP1 (SEP1), which has a broad spectrum and high activity against planktonic cells, was evaluated concerning its efficacy against *S. epidermidis* biofilms. The in vitro biofilm killing assays demonstrated a reduced activity of the phage. To understand the underlying factors impairing SEP1 inefficacy against biofilms, this phage was tested against distinct planktonic and biofilm-derived bacterial populations. Interestingly, SEP1 was able to lyse planktonic cells in different physiological states, suggesting that the inefficacy for biofilm control resulted from the biofilm 3D structure and the protective effect of the matrix. To assess the impact of the biofilm architecture on phage predation, SEP1 was tested in disrupted biofilms resulting in a 2 orders-of-magnitude reduction in the number of viable cells after 6 h of infection. The interaction between SEP1 and the biofilm matrix was further assessed by the addition of matrix to phage particles. Results showed that the matrix did not inactivate phages nor affected phage adsorption. Moreover, confocal laser scanning microscopy data demonstrated that phage infected cells were less predominant in the biofilm regions where the matrix was more abundant. Our results provide compelling evidence indicating that the biofilm matrix can work as a barrier, allowing the bacteria to be hindered from phage infection.

## 1. Introduction

Medical device-related infections are among the most common healthcare-associated infections (HAIs), causing increased morbidity and mortality on patients, which poses an abundant economic burden on healthcare services [1,2]. The considerable difficulty in the treatment of these infections stems mainly from the microorganisms’ ability to form biofilms. Biofilms can be briefly defined as communities of microorganisms attached to surfaces and surrounded by a self-produced polymeric matrix [3]. It is estimated that about 60–70% of HAIs are biofilm-related infections associated with implanted medical devices [4].

One of the key features of these three-dimensional microbial structures is their increased tolerance and resistance to antimicrobial therapies [5]. Bacterial biofilms are very difficult to treat with standard antibiotics for several reasons. The biofilm matrix acts as a barrier by itself, conferring protection to the cells [6]. Furthermore, biofilms are generally composed of bacterial cells that are in a wide range of physiological states [7]. Typically, biofilm cells display a genome-wide adaptation to that lifestyle, including downregulation of fundamental cell processes such as nucleic acid, protein, and cell wall biosynthesis [8]. Additionally, biofilms are composed of persister cells, which are a subset of antibiotic-tolerant cells within a bacterial population [9]. These cells are more prevalent in biofilms than on log-phase planktonic cultures, being associated with the recalcitrance of many chronic infections [10,11]. Another important reason for biofilm therapy inefficacy is related to the release of bacterial cells from the biofilm. Biofilm-released cells (BRCs), are associated with the development of endocarditis or bacteraemia [12,13,14]. Although it was initially thought that BRCs quickly revert to the planktonic phenotype [15], there is now evidence that these cells are distinct from planktonic cells, showing higher virulence potential [16].

*Staphylococcus epidermidis*, a ubiquitous organism that is currently regarded as an important nosocomial pathogen, is a major source of infections on implanted medical devices [17], mostly due to its ability to form biofilms [18]. Although the *S. epidermidis* biofilm matrix is composed by proteins, nucleic acids and lipids it is poly-*N*-acetylglucosamine (PNAG) that is regarded as its major component. This exopolysaccharide was previously reported to be involved in immune evasion by *S. epidermidis* and *S. aureus* [19]. *S. epidermidis* usually encodes specific antibiotic-resistance genes, namely against vancomycin and β-lactam antibiotics, which are used in first-line therapies against staphylococcal infections [20,21]. In an era of antibiotic-resistant bacteria emergence, alternatives to antibiotics are urgently needed. The use of bacteriophages (phages) to combat bacterial infections has been widely assessed, as several phages can target antibiotic-resistant bacteria [22]. Phages are viruses present in almost every ecological niche, being very specific to their bacterial hosts, and consequently harmless to human cells and human natural flora [23]. Phages are natural bacterial predators, and virulent phages represent a possible tool to treat bacterial infections [24,25]. In contrast to temperate phages, strictly virulent phages do not integrate their nucleic acids into the host chromosome, so their use for therapeutic purposes has been described as harmless to humans and animals [26,27]. Indeed, successful results have already been observed for the treatment of *Staphylococcus aureus* biofilm-associated infections [28,29]. However, only few studies have addressed the efficacy of *S. epidermidis* phages against biofilms [30]. In this study, we characterized the activity against *S. epidermidis* biofilms of a previously isolated *S. epidermidis*-specific *Sepunavirus* phiIBB-SEP1 (SEP1) phage, which was shown to be highly active against stationary-phase cells [31,32]. SEP1 also displayed a very low activity against biofilm cells compared to planktonic cells. Consequently, the factors behind the impaired SEP1 efficacy against biofilms were examined in this study.

## 2. Materials and Methods

### 2.1. Bacterial Strains and Culture Conditions

The biofilm-forming strain *S. epidermidis* 9142 was used in this study [33]. Bacteria were grown in Tryptic Soy Broth (TSB, Oxoid, Hampshire, UK), Tryptic Soy Agar (TSA; Oxoid), or in TSA soft overlays (TSB with 0.4% agar) at 37 °C. *S. epidermidis*-specific phage SEP1 was previously isolated and characterized [32].

### 2.2. Phage Production

SEP1 phage particles were produced as described before [32]. Briefly, 100 μL of a phage suspension at 10^6^ PFU·mL^−1^ was spread on a *S. epidermidis* 9142 lawn, using a paper strip. After 16 h incubation at 37 °C, full lysis was checked. Then, 3–4 mL of SM buffer [100 mM NaCl, 8 mM MgSO_4_, 50 mM Tris/HCl (pH 7.5), 0.002% (w/v) gelatin] were added to each plate. Subsequently, plates were agitated at 120 rpm in an orbital shaker (BIOSAN PSU-10i, Riga, Latvia) for 24 h at 4 °C. Thereafter, liquid and top agar were collected and centrifuged for 10 min, 10,000× *g*, 4 °C, and the supernatant filtered through a 0.22 μm cellulose acetate membrane (GE Healthcare, Little Chalfont, UK). Samples were stored at 4°C until further use.

### 2.3. Biofilm Formation

TSB (10 mL) was inoculated with one colony of *S. epidermidis* and incubated for 16 h in an orbital shaker (120 rpm, BIOSAN) at 37 °C. To establish mature biofilms, 2 μL of that culture were transferred to 96-well polystyrene plates (Orange Scientific, Braine-l’Alleud, Belgium) containing 198 μL of TSB supplemented with 1% (w/v) filtered glucose (TSBG) and incubated 24 h in an orbital shaker incubator (120 rpm, BIOSAN ES-20/60) at 37 °C. The samples were sonicated for 10 sec at 30% to eliminate clusters (Cole-Parmer^®^ 750 Watt Ultrasonic Homogenizer, 230 VAC, employing a 13-mm microtip) [34], and culturable cells determined using the microdrop method [35]. For biofilm matrix extraction experiments (Section 2.9), biofilms were formed according to the same procedure, but using 24-well polystyrene plates (Orange Scientific) in TSBG. Biofilms (*n* = 24) were then washed twice with saline solution (0.9% NaCl (w/v)), being further scraped and suspended in a total volume of 3 mL of saline solution.

### 2.4. Infection of Biofilms

Twenty-four h biofilms were infected as previously described [36], with some modifications. In brief, the supernatant of 24 h biofilms was removed and the biofilms washed twice with saline solution. Thereafter, 200 μL of phage suspension (~2 × 10^8^ PFU·mL^−1^ to obtain a Multiplicity of Infection (MOI) of 1) were added to each well. Microplates were incubated at 37 °C, 120 rpm, and samples were taken at 6 and 24 h post-infection. The number of culturable cells was determined as mentioned before (Section 2.3). Three independent experiments were performed in triplicate. Control experiments were performed by adding SM buffer instead of phage suspension.

### 2.5. Biofilm Biomass Quantification

The total biofilm biomass in each biofilm was determined by crystal violet assay as described before [33]. In brief, biofilms were washed twice with saline solution and then fixed with 250 μL of methanol (Merck, Kenilworth, NJ, USA). After 15 min, methanol was removed, and the plates air-dried. Thereafter, 250 μL of 1% crystal violet (*v*/*v*, Merck) were added to each well, incubated for 5 min at room temperature, and washed with tap water. Finally, 250 μL of 33% acetic acid (*v*/*v*, Merck) were added to each well to dissolve the stain, and the absorbance measured at 570 nm, in an enzyme-linked immunosorbent assay (ELISA) reader (Tecan, Maennedorf, Switzerland). Two independent experiments were performed in triplicate.

### 2.6. Infection of Disrupted Biofilms

To assess phage infection against disrupted biofilms, 24 h biofilms were washed twice with saline solution and were further slightly scraped from the surface using a micropipette tip [36]. After scraping, biofilms were infected using the conditions described for biofilm infections. In average ~5 × 10^8^ PFU·mL^−1^ of SEP1 was added to ~5 × 10^8^ colony-forming units (CFU).mL^−1^ of *S. epidermidis* cells to obtain a MOI of 1. Three independent experiments were performed in triplicate. Control experiments were performed by adding SM buffer instead of phage suspension.

### 2.7. Infection of Biofilm Released Cells (BRCs)

Twenty-four h biofilms were washed twice with saline solution, and TSBG was added to each well. Cells were grown at 37 °C, 120 rpm, and samples were taken after 3 h. Suspensions were centrifuged (5 min, 5000× *g*, 4 °C) and suspended in saline solution, sonicated for 10 sec at 30% to eliminate clusters, and optical density (OD_600nm_) was adjusted to approximately 0.2 (~2 × 10^8^ CFU.mL^−1^). Infection assays were performed using a MOI of 1 at 37 °C at 120 rpm. Samples were taken at 2, 4, and 8 h post-infection. Three independent experiments were performed in triplicate.

The samples were sonicated as described before to eliminate cell clusters without affecting cell viability [34], for 10 sec at 30%, and the number of culturable cells (CFU.mL^−1^) was quantified using the microdrop method, as described above. Three independent experiments were performed in triplicate. Control experiments were performed by adding SM buffer instead of phage suspension.

### 2.8. Infection of Persister Cells

Persister cells were obtained as described before [37]. Standard culture conditions, as described above, were used to prepare a culture of *S. epidermidis*. At 12 h, 50 µg·mL^−1^ of vancomycin were added to the bacterial culture and incubated for 48 h. Afterwards, cells were harvested (2 min, 14,000× *g*, 4 °C), and washed twice in 1 mL cold TSB.

The minimum inhibitory concentration (MIC) of the surviving cells was assessed as described by European Committee on Antimicrobial Susceptibility Testing (EUCAST), and the susceptibility pattern to vancomycin was kept [38]. Consequently, surviving cells were defined as persister cells. Cell suspensions were diluted with the supernatant of the remaining culture (centrifuged media used to grow the cells) to obtain an optical density at 600 nm (OD_600nm_) of approximately 0.5 (~5 × 10^8^ CFU.mL^−1^). Infection assays were performed as described above, using a MOI of 1. Samples were taken at 2, 4, and 8 h post-infection. Three independent experiments were performed in triplicate.

### 2.9. The Effect of the Biofilm Matrix on Phage Infectivity

The biofilm matrix from 24 h biofilms, formed as described above, was extracted as described before [39]. Briefly, after washing, biofilms were scraped, and the biofilm suspension was sonicated for 30 s at 30% (Cole Parmer, Hills, IL, USA) on ice, vortexed for 2 min, and centrifuged at 3000× *g*, for 10 min at 4 °C. Supernatants were collected and filtered through a 0.22 μm cellulose acetate filter (GE Healthcare). Protein and polysaccharide contents were determined, respectively, by bicinchoninic acid (BCA) protein assay (Thermo Scientific, Waltham, MA, USA) and Dubois method [40]. Matrix content quantifications were performed in triplicate.

To understand if the biofilm matrix impairs phage availability and viability, a total of 1 mL of biofilm matrix extracted as described above and containing 0.31 mg·mL^−1^ of proteins and 0.11 mg·mL^−1^ of polysaccharides, were added to 1 mL of a phage suspension containing ~2 × 10^8^ PFU·mL^−1^ (in SM buffer) at 37 °C. Phage titer was determined 2 h after incubation. Control experiments were performed with SM buffer instead of biofilm matrix. Three independent experiments were performed in triplicate.

To understand if the biofilm matrix interacts with phage particles inhibiting SEP1 efficacy against biofilm cells, adsorption assays were performed in the presence of biofilm matrix.

The adsorption efficiency of SEP1 to the host in the presence of the biofilm matrix was estimated with cells in the logarithmic phase (OD_600nm_ of approximately 0.6) [41]. A volume of 1 mL of matrix containing 0.31 mg·mL^−1^ of proteins and 0.11 mg·mL^−1^ of polysaccharides was added to 1 mL of the bacterial suspension (~6 × 10^8^ CFU.mL^−1^) and to 1 mL of the phage suspension at ~6 × 10^7^ PFU·mL^−1^ in order to obtain a MOI of 0.1. The mixture was incubated at 37 °C with shaking (120 rpm), and samples were collected after a total period of 5 min. Samples were further centrifuged at 16,000× *g* for 3 min, after which the phage titer remaining in the supernatant was determined. TSB was used as a non-adsorbing control in each assay, and the phage titer in the control supernatant was set to 100% [42]. Each assay was performed in duplicate and repeated three times.

### 2.10. Design of the LNA Probe

A probe targeting the mRNA encoding for the major capsid protein was designed essentially as described elsewhere [43] using SEP1 major capsid sequence (AGR48139) as target. The specificity of several potential target regions was analyzed by BLASTn [44].

The probe sequence was then adapted to include locked nucleic acid/2′-O-methyl-RNA nucleotides to be used in fluorescence in situ hybridization assays (LNA/2′,OMe-FISH), as previously described [45]. Theoretical melting temperatures and thermodynamic parameters of the different sequences were obtained using the RNA Chemistry Laboratory software (Poznan, Poland, http://rnachemlab.ibch.poznan.pl/calculator2.php).

Based on the GC percentage, presence, or absence of self-complementary structures and melting temperature, the following oligomer sequence was selected: 5′- TmAmGTmCmCAmUmGTmUmAAmCmA -3′ ′ (letters preceded by “m” indicate 2’-O-methyl-RNA nucleotides, while the other positions correspond to LNA nucleotides). The probe was designated SEP1p and was synthetized by Exiqon (Vedbaek, Denmark), attached to the TYE665 fluorochrome.

### 2.11. Spatial Organization of Phage-Infected Biofilms

To evaluate the spatial distribution of phage-infected cells, the LNA/2′OMe-FISH procedure in combination with confocal laser scanning microscopy (CLSM) analysis was performed directly on Thermanox™ coverslips (13 mm) (Nunc, Roskilde, Denmark) within a 24-well tissue culture plate. Biofilms were washed with saline solution and dried at 60 °C for 15 min to prevent detachment. Afterwards, biofilms were fixed in 100% methanol (Merck) for 20 min, in 4% (*v*/*v*) paraformaldehyde (Sigma-Aldrich), and 50% (*v*/*v*) ethanol (Panreac, Barcelona, Spain), for 15 min each at room temperature, and allowed to air dry.

Hybridization was performed essentially as previously described with some modifications [43]. Biofilms were covered with 20 μL of hybridization solution containing 900 mM NaCl (Panreac), 30% (*v*/*v*) formamide (Sigma), 20 mM Tris-HCl (pH 7.2; Sigma), 0.01% (wt/vol) sodium dodecyl sulfate (Bio-Rad, Berkeley, CA, USA) and 200 nM of LNA/2OMe probe. Samples were then covered with coverslips and incubated for 60 min at 56 °C. Coverslips were removed, and the slides were placed in a preheated (56 °C) washing solution containing 20 mM Tris base (pH 7.2, Sigma), 900 mM NaCl (Sigma) and 0.01% (*v*/*v*) sodium dodecyl sulfate (Sigma). Washing was performed for 30 min at 56 °C, and the slides were allowed to air dry and mounted with one drop of mounting oil (Olympus, Shinjuku City, Tokyo, Japan).

The images were acquired in CLSM (Olympus BX61, Model FluoView 1000). 4′,6-diamidino-2-phenylindole (DAPI) dye (Invitrogen, Carlsbad, CA, USA) was used for staining all nucleic acids (laser excitation line 405 nm and emissions filters BA 430–470, blue channel); wheat germ agglutinin (WGA) conjugated with Fluorescein isothiocyanate (FITC, Invitrogen) was applied to stain *N*-acetyl-D-glucosamine residues that comprise poly-*N*-acetylglucosamine (PNAG) [46] (laser excitation line 488 nm and emissions filters BA 505–605, green channel); and SEP1p probe targeting SEP1 (laser excitation line 635 nm and emissions filters BA 655–755, red channel) was used to stain *S. epidermidis* cells activity replicating the phage. Images were acquired with the program FV10-Ver4.1.1.5 (Olympus, Germany). Five surfaces of three independent replicates were observed in each CLSM experiment.

### 2.12. Statistical Analysis

The assays were compared using two-way analysis of variance (ANOVA) and Bonferroni post-test, using Prism 5 (GraphPad, La Jolla, CA, USA). Differences among conditions were considered statistically significant when *p* < 0.001.

## 3. Results

### 3.1. Phage Sepunavirus phiIBB-SEP1 (SEP1) Efficacy against Biofilms is not Significantly Pronounced

**Figure 1:**
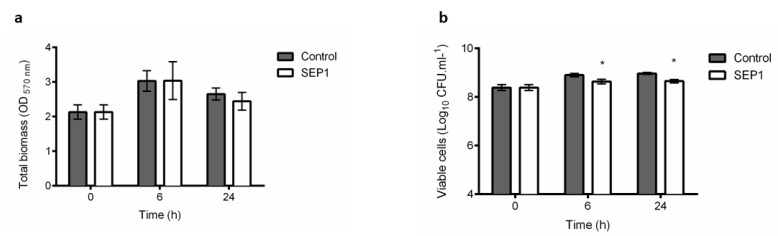
Characterization of the effect of *Sepunavirus* phiIBB-SEP1 (SEP1) on 24 h *S. epidermidis* 9142 biofilms, using a MOI 1. (**a**) Effect on the total biomass of biofilms was assessed by crystal violet. staining measured at an optical density OD_570_; (**b**) effect on the total number of viable cells was assessed by colony-forming unit (CFU) counting. The values represent the mean plus and minus standard deviation of two independent experiments performed in triplicate. Statistical differences (*p* < 0.001) between control and SEP1-treated cells (*) were determined by two-way repeated-measures analysis of variance (ANOVA) with Bonferroni post hoc test.

Biofilms are characterized by a heterogeneous population of cells with different physiological conditions. SEP1′s efficacy to infect biofilms was tested (Figure 1). After phage infection of 24 h biofilms, there was no significant reduction of the total biofilm biomass (Figure 1a), despite the slight decrease (0.3 orders-of-magnitude) observed on the total number of culturable cells during infection compared to untreated biofilms (Figure 1b).

SEP1 is highly specific for *S. epidermidis* and presents good infective properties such as low latency time and ability to significantly reduce the optical density of several strains, as previously shown [32]. Moreover, we also showed that SEP1 has the rare feature of infecting and replicating efficiently in stationary phase cells [31]. Due to the inefficacy of this phage against 24 h biofilms, a more in-depth analysis was performed to evaluate SEP1′s effect in different populations of biofilm-related cells.

### 3.2. SEP1 Can Control Biofilm-Released Cells (BRCs)

**Figure 2:**
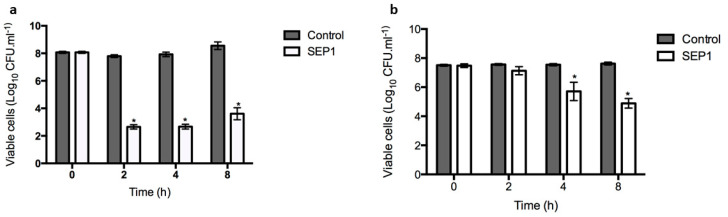
SEP1 phage infection of planktonic *S. epidermidis* 9142 planktonic cultures. (**a**) biofilm-released cells and (**b**) persister cells were infected with SEP1 using a MOI of 1. Data was assessed by CFU counting and the values represent the mean plus and minus of three independent experiments performed in triplicate. Statistical differences (*p* < 0.001) between control and SEP1-treated cells (*) were determined by two-way repeated-measures analysis of variance (ANOVA) with Bonferroni post hoc test.

Due to the increasing clinical relevance attributed to the *S. epidermidis* BRCs, which have a particular phenotype of presenting higher tolerance to antibiotics than planktonic and biofilm cells [47], SEP1′s efficiency against this bacterial population was tested (Figure 2a). BRCs were obtained using a fed-batch system in the presence of culture medium and under agitation. SEP1 was shown to infect *S. epidermidis* BRCs after 2 h of infection, reducing about 5 orders-of-magnitude the number of culturable cells, which was maintained until 4 h of infection (Figure 2a). However, after 8 h there was a slight significant increase in the total number of viable cells that might be related with the development of phage resistant mutants. Despite the known increased tolerance of BRCs to antibiotics, it was demonstrated herein that this type of cell remains susceptible to the phage.

### 3.3. SEP1 Can Infect Persister Cells

Persister cells are a subset of the biofilm population, which are tolerant to antibiotic killing, being responsible for the recalcitrance of infections [9]. *S. epidermidis* persister cells were obtained after contact with vancomycin for 48 h. The effect of SEP1 against persister cells is visible in Figure 2b. In general, SEP1 demonstrated a poor reduction ability against persister cells in the first 2 h, achieving a better reduction of 2 orders-of-magnitude at 4 h. After 8 h, SEP1 reduced by approximately 3 orders-of-magnitude the number of *S. epidermidis* culturable cells.

### 3.4. Infection of Disrupted Biofilms

**Figure 3:**
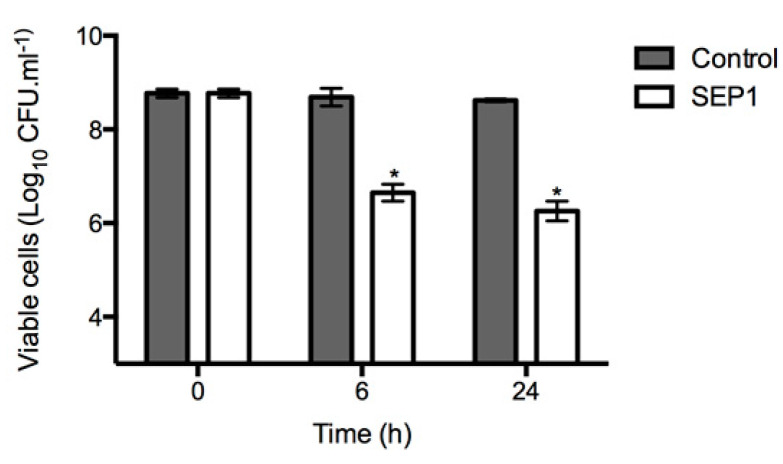
SEP1 phage infection of 24 h *S. epidermidis* 9142 disrupted biofilms, using a MOI 1. Data was assessed by CFU counting and the values represent the mean plus and minus of three independent experiments performed in duplicate. Statistical differences (*p* < 0.001) between control biofilms and SEP1-treated biofilms (*) were determined by two-way repeated-measures analysis of variance (ANOVA) with Bonferroni post hoc test.

**Figure 4:**
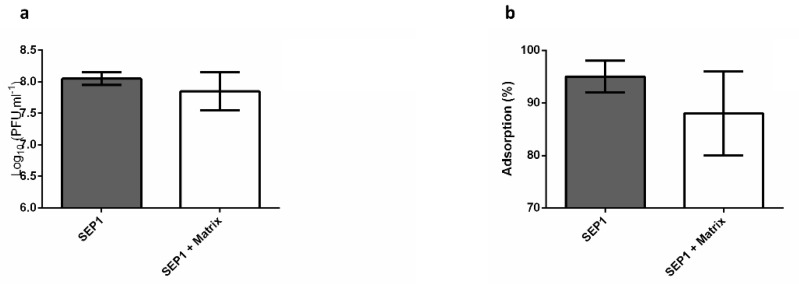
Effect of biofilm matrix on SEP1 efficiency. (**a**) Phage infectivity in a biofilm matrix suspension after 2 h of incubation at 37 °C. Data was assessed by PFU counting and the values represent the mean plus and minus of three independent experiments performed in triplicate; (**b**) phage adsorption in the presence of biofilm matrix. Data was assessed by PFU counting and the values represent the mean plus and minus of two independent experiments performed in triplicate.

Although not equally effective against all types of planktonic cells tested, since SEP1 is able to infect *S. epidermidis* cells in different metabolic/physiologic states, we hypothesized that its poor activity against biofilms must be related to the biofilm structure itself. To test this hypothesis, we disturbed the biofilms, disintegrating their 3-D structure. In all sampled time points, an evident increase in phage efficacy was observed in disrupted biofilms in comparison with intact biofilms (Figure 3). The mechanical effect of scraping did not lead to complete detachment of the biofilm cells since the number of culturable cells was maintained during the entire experiment (see control columns in Figure 4). SEP1 caused significantly higher reductions on the number of culturable cells in disrupted biofilms after 6 h (approximately 2 orders-of-magnitude) and 24 h (approximately 2.5 orders-of-magnitude) than in intact biofilms (Figure 1b), respectively.

### 3.5. The Effect of the Biofilm Matrix on Phage Efficiency

The biofilm matrix was extracted from biofilm cells and put in contact with SEP1 for 2 h at 37 °C. The results demonstrated that the biofilm matrix did not impair phage’s infectivity, as no significant differences (*p* > 0.05) were detected in the phage titer between matrix containing experiments and the negative control (Figure 4a).

Furthermore, to understand if the biofilm matrix can interact with the phage particles, limiting their access to host cells, adsorption assays were performed in the presence of matrix. Our results suggest that there is no interference of the biofilm matrix on planktonic cell adsorption as no significant differences (*p* > 0.05) were obtained between negative control and biofilm matrix-containing samples (Figure 4b).

### 3.6. Spatial Organization of Phage-Infected Biofilms

**Figure 5:**
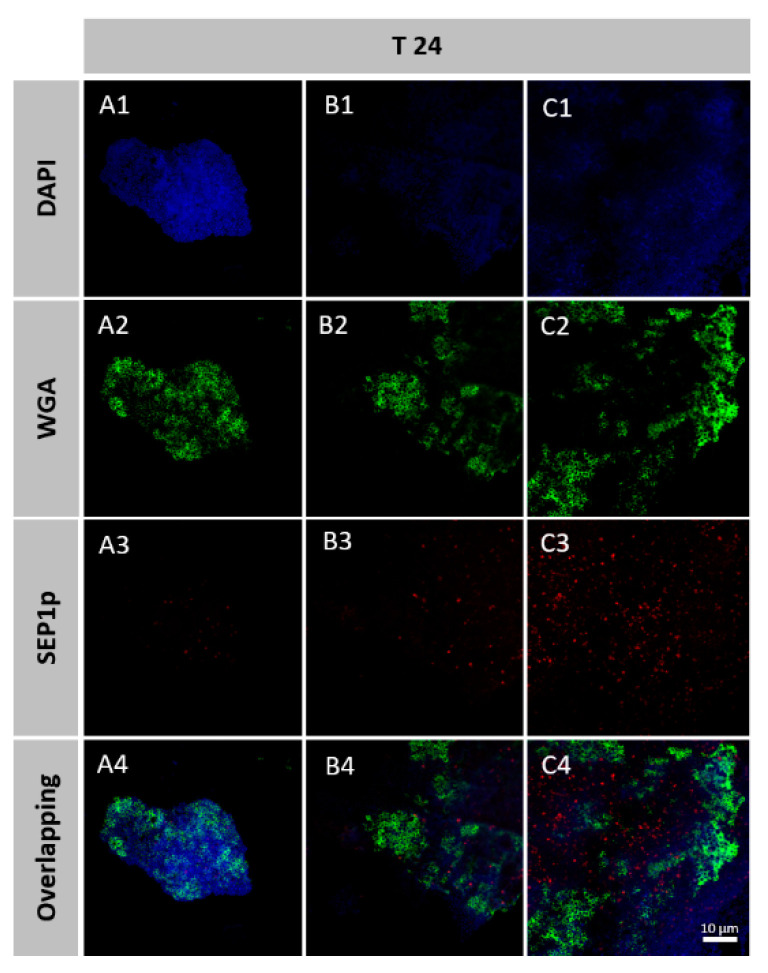
Confocal laser scanning microscopy (CLSM) images of *S. epidermidis* 9142 biofilm after phage treatment (**A**–**C**). The blue fluorescence corresponds to *S. epidermidis* cells, the green fluorescence corresponds to the matrix and red fluorescence shows the phage staining using the phage probes SEP1p. The bottom image present the overlap of the three channels discriminating the infected cells, non-infected cells and the matrix.

A probe was developed to detect the location of the biofilm cells that are infected by phage SEP1. Biofilms were formed directly on Thermanox™ coverslips and infected with SEP1 for 24 h. Biofilms were further fixed, stained, and observed by CLSM. CLSM in conjugation with the probe and two different dyes was used to differentiate bacterial cells from phage-infected cells and major matrix component PNAG (Figure 5).

Multiplex experiments with phage probes and the two dyes discriminate between infected and non-infected bacterial populations and PNAG. After 6 h and 24 h of infection, it was possible to observe areas with infected and non-infected cells along with the biofilm. The intense red signal of the SEP1p probe, which allowed the detection of phages replicating within *S. epidermidis* biofilms, demonstrated that phage infected cells seem to be predominantly located in regions with lower amounts of PNAG (Figure 5).

## 4. Discussion

The interest in alternatives to antibiotic-based therapies for the treatment of biofilm-associated infections has increased in the last decades, with phages re-emerging as promising tools to combat infections, particularly those caused by antibiotic-resistant pathogens [48]. Although there are several reports on the efficacy of phages against planktonic cells in the exponential growth phase, few studies have focused on their use against stationary-phase cells [31,49,50,51,52] and biofilm populations (reviewed in [53]). Previous results with a *S. epidermidis*-specific phage that belongs to the *Sepunavirus* genus (SEP1) showed that it displays a broad host range being active against stationary-phase cells [31]. Despite these two characteristics, SEP1 application as an anti-biofilm agent was unsuccessful, with less than 0.5 orders-of-magnitude decrease on the number of culturable cells observed (Figure 1b). The poor efficacy of staphylococcal myoviruses towards biofilms has already been described for instance for the polyvalent *Staphylococcus* phage K [30].

To understand the underlying causes of SEP1′s inefficacy towards biofilms, phage interaction with different biofilm-associated cell populations was assessed. Interestingly, this phage showed an increased ability to kill BRC and persister cell populations in comparison with the entire biofilm. Furthermore, we have previously demonstrated that this phage has a remarkable ability to kill planktonic bacterial populations in the late stationary phase [31]. It is generally accepted that stationary phase cells are usually more tolerant to antibiotics and disinfectants than log phase planktonic cells. The efficiency of SEP1 against stationary phase cells is, therefore, of extreme value since it can target this population that most antibiotics fail to address. Taking into account that late stationary cultures have a high content of cells in a dormant state [54], it is not that surprising that this phage can infect persister cells. Persister cells emerge due to a state of dormancy, in which cells are metabolically inactive, being involved in the failure of antibiotic therapies [55]. This cell population was the least affected by SEP1, yet still, and unlike many antibiotics [37,54], this phage reduced by approximately 3 orders-of-magnitude the amount of culturable cells after an infection period of 8 h. To our knowledge, and despite the transience of persister cells, this cell population was only targeted before in *S. aureus* [56], and such a reduction can be therapeutically valuable since antibiotics are inefficient against this population.

Biofilm dispersion is one of the less-studied biofilm cycle stages. BRCs are described as being more virulent due to their increased tolerance to antibiotics than planktonic or biofilm cells, as evaluated by viable cell counts [47]. In that study, *S. epidermidis* 9142 BRCs were less affected by tetracycline than biofilm, stationary-phase, and exponential phase cells. To understand if BRCs represent an intermediate phenotype and if SEP1 holds the potential to be further used in clinical practice to prevent biofilm development, the efficacy of this phage was assessed against this cell population. Interestingly, SEP1 significantly reduced BRCs in about 5 orders-of-magnitude, surpassing the antimicrobial effect of any tested antibiotics in this strain (tetracycline, rifampicin, vancomycin) [47].

Overall, the results presented here show that SEP1 efficiently reduced different biofilm populations, and therefore its reduced efficacy against intact biofilms must be related with their complex architecture [57]. Although such hypothesis has been frequently suggested [58,59], no detailed studies have addressed the importance of matrix in phage-biofilm interaction. To test this hypothesis, an assay was developed in which the biofilm architecture was mechanically disrupted, and the partially disrupted biofilms were further challenged with SEP1. Accordingly, using the same infection conditions as those used against intact biofilms, it was observed that cells from disrupted biofilms were more susceptible to SEP1, which might be a consequence of an enhanced contact between phage and biofilm cells. However, this reduction was still inferior to that occurring on planktonic cells, namely BRCs. Although not in its native form, we observed on CLSM that disrupted biofilms still contain small clusters of biofilms, suggesting a possible role of the matrix in hindering phage efficacy. To understand if the biofilm matrix contained some active compounds that inhibited phage particles, phages were placed in contact solely with the extracted biofilm matrix. After 2 h of contact, it was possible to observe that phage particles were not inactivated by the biofilm matrix (Figure 4a), suggesting that SEP1 is tolerant to some *S. epidermidis* matrix compounds, namely proteases [60]. Moreover, our results demonstrated that phage adsorption to the host in the presence of biofilm matrix was not affected (Figure 4b). These results suggest that there is no irreversible adsorption of SEP1 to any of the biofilm matrix compounds.

Lastly, phage-infected cells in intact biofilms were discriminated using a LNA/2′,OMe-FISH method associated with CLSM. During biofilm maturation, matrix accumulation increases [34] as observable by a very thick amount of PNAG visibly external to the cells (Figure 5) and also by an increase of biomass (Figure 1a). With several microscopy observations, we noticed that phage-infected cells only appeared in certain regions of the biofilm, mainly in the regions that have lower amounts of PNAG (Figure 5). It was previously reported that several factors might influence phage diffusion throughout the biofilm structure, namely the applied phage titer, the amount of attached biomass, strain susceptibility, phage entrapment in the extracellular matrix, and phage inactivation [61]. Herein, the results suggest that although SEP1 can migrate throughout the biofilm, it demonstrated more difficulty in reaching the biofilm areas exhibiting higher PNAG content. In another study using simulations, it was hypothesized that the biofilm matrix is essential in phage/biofilm interactions by altering phage mobility and by blocking access to new hosts [62]. This phenomenon could be circumvented by the presence of matrix-degrading enzymes encoded on phage genomes, named phage depolymerases [63]. However, SEP1 plaque morphology was not evidenced the presence of an increasing halo and when the genome of SEP1 was previously analyzed, no phage depolymerases were found [32]. Consequently, our results provide evidence that it is the *S. epidermidis* complex biofilm architecture that limits SEP1 diffusion and ultimately its efficacy.

## 5. Conclusions

This study reveals that the phage SEP1 inefficiency against biofilms was related to the biofilm 3D structure and architecture. The matrix, in particular, limits phage access to the biofilm cells acting as an evasion mechanism to phage predation.

The fact that SEP1 efficiently infects planktonic cells with different phenotypes suggests that this phage might be a promising candidate for therapy, namely in the treatment of *S. epidermidis* biofilm-related infections. This treatment could be undertaken in association with debridement or the phage can be genetically modified to express matrix-degrading enzymes.
